# Drug repositioning for SARS-CoV-2 by Gaussian kernel similarity bilinear matrix factorization

**DOI:** 10.3389/fmicb.2022.1062281

**Published:** 2022-12-05

**Authors:** Yibai Wang, Ju Xiang, Cuicui Liu, Min Tang, Rui Hou, Meihua Bao, Geng Tian, Jianjun He, Binsheng He

**Affiliations:** ^1^School of Information Engineering, Changsha Medical University, Changsha, China; ^2^Academician Workstation, Changsha Medical University, Changsha, China; ^3^School of Life Sciences, Jiangsu University, Zhenjiang, Jiangsu, China; ^4^Geneis (Beijing) Co., Ltd., Beijing, China; ^5^Qingdao Geneis Institute of Big Data Mining and Precision Medicine, Qingdao, China; ^6^School of Pharmacy, Changsha Medical University, Changsha, China; ^7^Key Laboratory Breeding Base of Hunan Oriented Fundamental and Applied Research of Innovative Pharmaceutics, Changsha Medical University, Changsha, China

**Keywords:** SARS-CoV-2, drug repositioning, bilinear matrix factorization, molecular docking, machine learning

## Abstract

Coronavirus disease 2019 (COVID-19), a disease caused by severe acute respiratory syndrome coronavirus 2 (SARS-CoV-2), is currently spreading rapidly around the world. Since SARS-CoV-2 seriously threatens human life and health as well as the development of the world economy, it is very urgent to identify effective drugs against this virus. However, traditional methods to develop new drugs are costly and time-consuming, which makes drug repositioning a promising exploration direction for this purpose. In this study, we collected known antiviral drugs to form five virus-drug association datasets, and then explored drug repositioning for SARS-CoV-2 by Gaussian kernel similarity bilinear matrix factorization (VDA-GKSBMF). By the 5-fold cross-validation, we found that VDA-GKSBMF has an area under curve (AUC) value of 0.8851, 0.8594, 0.8807, 0.8824, and 0.8804, respectively, on the five datasets, which are higher than those of other state-of-art algorithms in four datasets. Based on known virus-drug association data, we used VDA-GKSBMF to prioritize the top-k candidate antiviral drugs that are most likely to be effective against SARS-CoV-2. We confirmed that the top-10 drugs can be molecularly docked with virus spikes protein/human ACE2 by AutoDock on five datasets. Among them, four antiviral drugs ribavirin, remdesivir, oseltamivir, and zidovudine have been under clinical trials or supported in recent literatures. The results suggest that VDA-GKSBMF is an effective algorithm for identifying potential antiviral drugs against SARS-CoV-2.

## Introduction

Caused by severe acute respiratory syndrome coronavirus 2 (SARS-CoV-2), a new infectious disease called coronavirus disease 2019 (COVID-19) has caused a big pandemic worldwide since 2019 (Eurosurveillance editorial team, 2020; Cheng et al., 2021a; Zhang et al., 2021). SARS-CoV-2 can transmit by human-to-human contacts, and is currently spreading rapidly to more than 400 countries around the world, causing millions of deaths (Coronaviridae Study Group of the International Committee on Taxonomy of V, 2020; Li et al., 2020; Cohain et al., 2021). Thus, SARS-CoV-2 seriously threatens human life and health as well as the development of world economy (Wu et al., 2020; Zhou P. et al., 2020; Zhu et al., 2020; Cheng et al., 2021b), and it is critical to find effective measures to prevent the transmission and fight against this virus.

One effective way to prevent the transmission of a virus is through vaccination. However, viruses like SARS-CoV-2 and influenzas are under rapid genetic and antigenic evolution, especially in their spike proteins (Yao et al., 2017; Zhang et al., 2017), which will make the vaccine less effective. Another method is to develop specific drug against the viruses. However, traditional methods to develop new drugs usually take years and cost tens of millions of dollars (Novac, 2013). With the development of various computational algorithms for mining intrinsic associations in biomedical data (Zhang et al., 2019; Xu et al., 2020a; Liu et al., 2021; Xiang et al., 2021a, 2022b; He et al., 2022; Yang et al., 2022), drug repositioning has become an effective way of exploring new uses for approved drugs, since it can significantly reduce the time and cost in the development of drugs (Liu et al., 2016, 2020; Yang J. et al., 2020; Zhu et al., 2021).

There are a few studies to prioritize approved drugs against SARS-CoV-2. For example, Zhou et al. proposed a KATZ method to probe antiviral drugs against SARS-CoV-2 through virus-drug association prediction (Zhou L. et al., 2020). More recently, Tang et al. prioritized drugs for COVID-19 through an indicator regularized non-negative matrix factorization method (Tang et al., 2020). Peng et al. collected an antivirial drug database and minied it to repurpose drugs aginst SARS-CoV-2 (Peng et al., 2020; Zhou L. et al., 2020). Wang et al. predicted anti-SARS-COV-2 drugs by bound nuclear norm regularization (Wang et al., 2021). Meng et al. builded the human drug virus database and identified anti-SARS-COV-2 drugs by similarity constrained probabilistic matrix factorization (Lu et al., 2021; Meng et al., 2021; Parsza et al., 2021). Shen et al. prioritized anti-SARS-CoV-2 drugs by combining an unbalanced bi-random walk and Laplacian regularized least squares (Shen et al., 2022). Though these methods achieved relatively good prediction performance in cross-validation and literature mining, the accuracy of prediction is yet to be improved and a more robust validation method is needed for further wet-lab experiments. Therefore, in this study, we collected the data of well-studied viruses that are similar to SARS-CoV-2 and their known antiviral drugs, forming a virus-drug association matrix (VDA). Then, we proposed a novel method for exploring potential virus-drug associations of SARS-CoV-2 by using Gaussian kernel similarity bilinear matrix factorization (VDA-GKSBMF).

The rest of the work is organized as follows. First, we collect five datasets and propose the details of the VDA-GKSBMF method for predicting potential virus-drug associations of SARS-CoV-2. Then, we study the effectiveness of the method by the 5-fold cross-validation experiments and compare VDA-GKSBMF with other state-of-art algorithms. Based on known virus-drug association data, we use VDA-GKSBMF to prioritize top-10 candidate antiviral drugs that are most likely to fight against SARS-CoV-2, and then evaluate the molecular binding activity between predicted antiviral drugs and SARS-CoV-2 spike protein (Gralinski, 2020) or human ACE2 (Zhao et al., 2020), to confirm whether the top-10 drugs are to be molecularly docked with the virus spikes protein or human ACE2. We also explore literatures to check if the top predicted drugs are under clinical trials or experiments against SARS-CoV-2.

## Materials and methods

**Figure 1:**
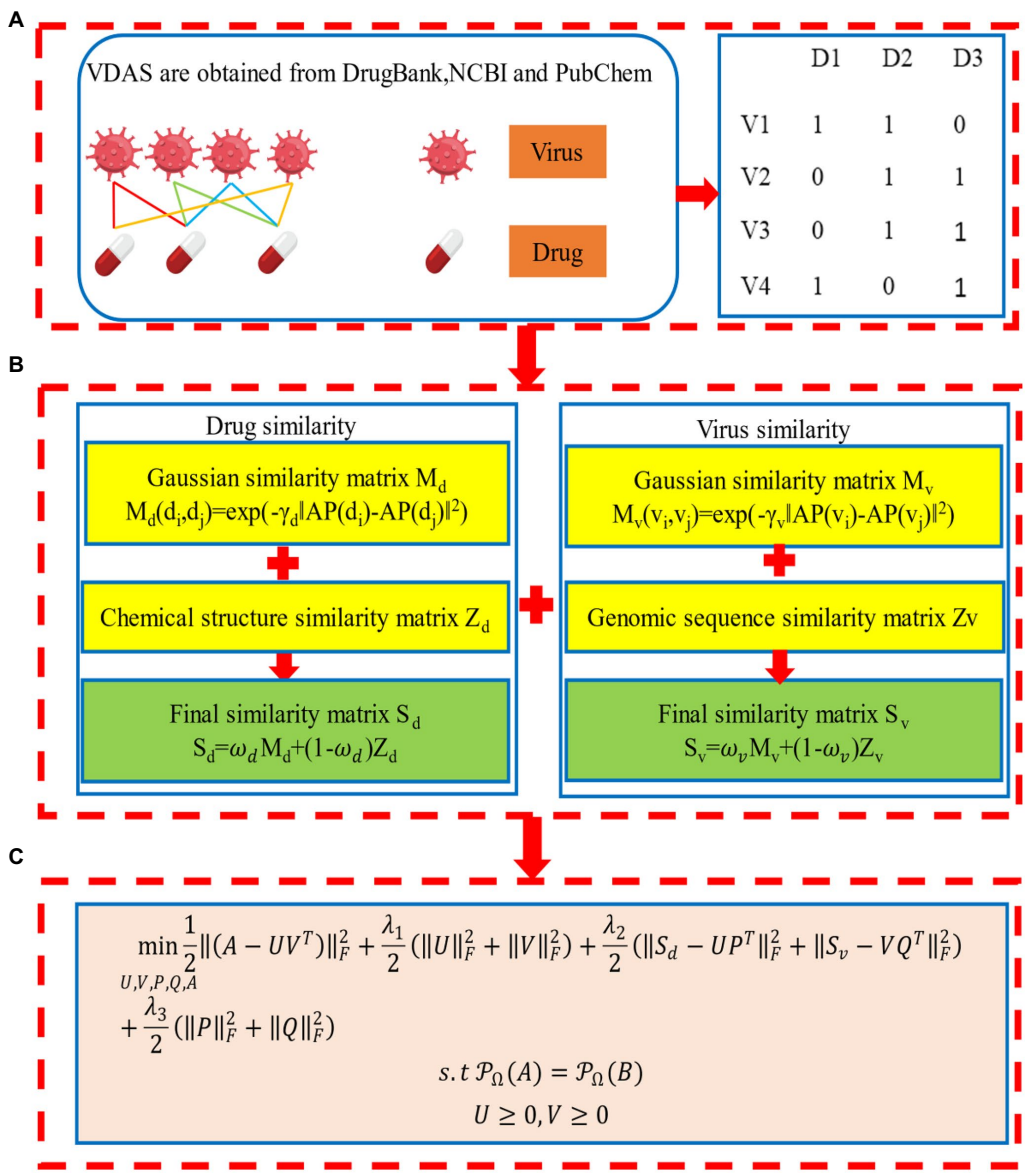
Workflow of Gaussian kernel similarity bilinear matrix factorization (VDA-GKSBMF). **(A)** Virus–drug association network and its association matrix. **(B)** Drug–drug similarity matrix and Virus–virus similarity matrix. **(C)** The model of VDA-GKSBMF.

The overall workflow of the method is illustrated in Figure 1. We first introduce the datasets in this study, and then describe the details of the VDA-GKSBMF method for drug repositioning of SARS-CoV-2, including the construction of virus–drug heterogeneous network and the VDA-GKSBMF model, along with the alternating direction method of multipliers (ADMM) for solving the model to fill out unknown associations in virus–drug matrix.

### Materials

To identify potential VDAs involving SARS-COV-2, we collect five datasets. There is Virus similarity matrix, drug similarity matrix, and VDA matrix in each dataset. Viruses are similar to SARS-CoV-2, small-molecule drugs and VDAs between them from the DrugBank (Wishart et al., 2018), PubChem (Kim et al., 2016), and NCBI (Wheeler et al., 2004) databases (see Table 1 for details).

**Table 1:** The statistics of datasets.

Datasets	No. of viruses	No. of drug	No. of VDAS	Sparsity
Dataset1	12	78	96	89.7%
Dataset2	69	128	770	91.3%
Dataset3	34	203	407	95.0%
Dataset4	34	210	437	93.9%
Dataset5	34	219	455	93.9%

These VDAs are represented by a VDA matrix B_m × n_, where B_dv_ = 1 if the d-th drug is associated with the v-th virus, otherwise, B_dv_ = 0. This forms a virus-drug association network, which can be denoted as a bipartite graph $G\left(V,D,E\right)$, where $E\left(G\right)=\left\{e_{ij}\right\}\subseteq V\times D$ contains edges representing known associations between viruses and drugs.

For viruses, we obtain the sequence-based similarities between viruses that are calculated by MAFFT (Katoh and Toh, 2008). For drugs, we obtain the chemical structure-based similarity scores between drugs by RDKit (Landrum, 2014), where chemical structures of drugs are obtained from the DrugBank database (Wishart et al., 2018). The details are shown in Table 1.

### Methods

#### Drug similarity matrix

Considering that drugs with common associated viruses may be similar, we denote the Gaussian association profile (AP) of drug $d_{i}$ by $AP\left(d_{i}\right)$, i.e., the i-th row of the VDA matrix B, which is a binary vector encoding the associations between this drug and viruses in the VDA matrix. Then, we calculate the similarity $M_{d}\left(d_{i},d_{j}\right)$ between two drugs $d_{i}$ and $d_{j}$ based on association profiles of drugs by,


$$M_{d}\left(d_{i},d_{j}\right)=\exp\left(-\gamma_{d}∥AP\left(d_{i}\right)-AP\left(d_{j}\right){∥}^{2}\right)$$

where $\gamma_{d}=\gamma \prime_{d}$/($\frac{1}{m}\sum_{k=1}^{m}∥AP\left(d_{k}\right){∥}^{2}$) is the normalized core band-width based on bandwidth parameter $\gamma \prime_{d}$, and m denotes the number of drugs.

Then, we obtain the chemical structure (CS)-based similarity between drugs calculated by RDKit (Landrum, 2014), which is denoted as $Z_{d}$. Finally, we generate the drug–drug similarity matrix (DDS) by,


$$S_{d}=\omega_{d}M_{d}+\left(1-\omega_{d}\right)Z_{d}\text{,}$$

where $\omega_{d}\in \left[0,1\right]$ balances the contribution of the CS-based and AP-based drug similarity matrices. This forms a drug–drug network with edges weighted by the pairwise drug similarity scores.

#### Virus similarity matrix

Considering that viruses with common associated drugs may be similar, in the same way, we denote the Gaussian association profile (AP) of virus $v_{a}$ by $AP\left(v_{a}\right)$, i.e., the a-th column of the VDA matrix B, which is a binary vector encoding the associations between this virus and drugs in the VDA matrix. We calculate the AP-based similarity $M_{v}\left(v_{a},v_{b}\right)$ between two viruses by,


$$M_{v}\left(v_{a},v_{b}\right)=\exp\left(-\gamma_{v}∥AP\left(v_{a}\right)-AP\left(v_{b}\right){∥}^{2}\right)\text{,}$$

where $\gamma_{v}=\gamma \prime_{v}/(\frac{1}{n}\sum_{k=1}^{n}∥AP\left(v_{k}\right){∥}^{2}$), and n denotes the number of viruses.

Then, we obtain the sequence (SQ)-based similarity matrix calculated by MAFFT (Katoh and Toh, 2008), which is denoted as $Z_{v}$. Finally, the virus-virus similarity matrix (VVS) is calculated by,


$$S_{v}=\omega_{v}M_{v}+\left(1-\omega_{v}\right)Z_{v}\text{,}$$

where $\omega_{v}\in \left[0,1\right]$ balances the contribution of the SQ-based and AP-based virus similarity matrices. This forms a virus-virus network with edges weighted by the pairwise virus similarity scores.

#### Constructing heterogeneous network

To make use of information in the above DDS, VVS, and VDA matrices, we integrate them to construct a heterogeneous virus–drug network, by connecting the virus–virus network and drug–drug network through virus–drug associations. In the heterogeneous network, there are a set of m viruses $V=\left\{v_{1},v_{2},v_{3},\ldots ,v_{m}\right\}$ and a set of n drugs $D=\left\{d_{1},d_{2},d_{3},\ldots ,d_{n}\right\}$; the edge between drugs $\left(d_{i},d_{j}\right)$ is weighted by the score $S_{d}\left(d_{i},d_{j}\right)$ in the DDS matrix, the edge between viruses $\left(v_{a},v_{b}\right)$ is weighted by the score $S_{v}\left(v_{a},v_{b}\right)$ in the VVS matrix, and the edge between drug $d_{i}$ and virus $v_{a}$ denotes the existence of association between them.

The VDA matrix B is extremely sparse due to the rarity of known virus–drug associations, where 1/0 denotes known/unknown virus–drug associations, respectively. We would like to fill out the missing values in the matrix as scores to predict unknown VDAs. The integration of information of DDSs, VVSs, and known VDAs into the heterogeneous network will benefit the discovery of unknown VDAs due to the intrinsic correlation among drugs and viruses.

#### VDA-GKSBMF model to predict virus–drug associations

To predict potential virus-drug associations of COVID-19, we define the VDA prediction as a problem of completing virus-drug matrix in a heterogeneous virus-drug network, and explore potential VDAs of COVID-19 by Gaussian kernel similarity bilinear matrix factorization (Yang M. et al., 2020; called as VDA-GKSBMF).

Matrix factorization is an effective method, which intends to calculate an optimal approximation to the target matrix by decomposing it into two low-rank matrices. In a word, the mathematical model of matrix factorization is formulated as


(1) $$\min_{U,V}B-U{V^{T}}_{F}^{2}\text{,}$$

where $B\in {\mathbb{R}}^{n\times m}$ is the given incomplete matrix with n drugs and m viruses, $U\in {\mathbb{R}}^{n\times k}$ and $V\in {\mathbb{R}}^{m\times k}$ are the indicator feature matrices of B and k is the subspace dimensionality [k $\ll \min\left(n,m\right)$], ${\;}_{∥.∥F}$ denotes the Frobenius norm. Many algorithms have been designed to provide numerical solutions for the above model or alternative forms. However, compared with other algorithms, the classic ADMM algorithm is superior to solving our proposed matrix factorization model.

The elements in the association matrix B are either 0 or 1. Thus, the predicted values in the un-known entries are expected to be in the interval of [0, 1], where a predicted value closer to 1 indicates that this is likely to be an indication and vice versa. Nevertheless, in the above matrix completion model, the entries in the completed matrix can be any real value in (−∞, +∞).

Moreover, based on the assumption that similar drugs share similar molecular pathways to treat similar viruses, the underlying factors that determine drug-virus associations are highly correlated. Since B is extremely rare and low rank, usually less than 1% of known associations are present, while the rest of the elements are unknown. Therefore, the error term is only computed on items with known associations. At the same time, Tikhonov regularization terms are often used to avoid overfitting. To achieve this, the matrix factorization model can be expressed as,


(2) $$\underset{U,V}{\;\min\frac{1}{2}}P\Omega ∥\left(B-UV^{T}\right){∥}_{F}^{2}+\frac{\lambda_{1}}{2}\left(∥U{∥}_{F}^{2}+∥V{∥}_{F}^{2}\right)\text{,}$$

where Ω is a set containing index pairs $\left(i,j\right)$ of all known entries in B and $P_{\Omega }$ is the projection operator onto Ω,$\lambda_{1}$ is regularization parameter. However, the above objective function does not involve a large amount of prior information about viruses and drugs, such as disease similarity and drug similarity. Since U and V are matrices containing potential eigenvectors of drugs and viruses, given a drug similarity matrix $Z_{d}$ and a virus similarity matrix $Z_{v}$, $UU^{T}$ and $VV^{T}$ are expected to match $S_{d}$ and $S_{v}$, respectively. Therefore, model (2) is described as follows:


(3) $$\begin{array}{l} \underset{U,V}{\min\frac{1}{2}}∥P_{\Omega }\left(B-UV^{T}\right){∥}_{F}^{2}+\frac{\lambda_{1}}{2}\left(∥U{∥}_{F}^{2}+∥V{∥}_{F}^{2}\right)\\ \;+\frac{\lambda_{2}}{2}\left(∥Z_{d}-UU^{T}{∥}_{F}^{2}+∥Z_{v}-VV^{T}{∥}_{F}^{2}\right) \end{array}$$

Model (3) deals with a single drug and virus similarity measure. Here, in order to integrate the Gaussian kernel similarity measure, we propose the VDA-GKSBMF model, which is expressed as follows:


(4) $$\begin{array}{l} \underset{U,V,P,Q,A}{\;\min\frac{1}{2}}∥\left(A-UV^{T}\right){∥}_{F}^{2}+\frac{\lambda_{1}}{2}\left(∥U{∥}_{F}^{2}+∥V{∥}_{F}^{2}\right)\\ \;+\frac{\lambda_{2}}{2}\left(∥S_{d}-UP^{T}{∥}_{F}^{2}+∥S_{v}-VQ^{T}{∥}_{F}^{2}\right)\\ \;+\frac{\lambda_{3}}{2}\left(∥P{∥}_{F}^{2}+∥Q{∥}_{F}^{2}\right)\; \end{array}$$

$$s.t\;P_{\Omega }\left(A\right)=P_{\Omega }\left(B\right)$$

U≥0,V≥0,

where $S_{d}$ and $S_{v}$ are matrices concatenating Gaussian kernel similarity measure of drug and virus, and $\lambda_{1}$, $\lambda_{2}$, and $\lambda_{3}$ are balancing parameters. A is an auxiliary matrix for facilitating optimization. The approximation of similarity matrix $S_{d}$ and $S_{v}$ are constructed based on characteristic matrices U and V, where P and Q are potential characteristic matrices representing drug similarity and virus similarity, respectively. We solve model (4) by ADMM framework. Introducing two riving matrices X and Y, model (4) is transformed into


(5) $$\begin{array}{l} \underset{U,V,P,Q,X,Y,A}{\;\min\frac{1}{2}}∥\left(A-UV^{T}\right){∥}_{F}^{2}+\frac{\lambda_{1}}{2}\left(∥U{∥}_{F}^{2}+∥V{∥}_{F}^{2}\right)\\ \;+\frac{\lambda_{2}}{2}\left(∥S_{d}-UP^{T}{∥}_{F}^{2}+∥S_{v}-VQ^{T}{∥}_{F}^{2}\right)\\ \;+\frac{\lambda_{3}}{2}\left(∥P{∥}_{F}^{2}+∥Q{∥}_{F}^{2}\right) \end{array}$$

$$s.t\;P_{\Omega }\left(A\right)=P_{\Omega }\left(B\right)$$

U=X,V=Y

X≥0,Y≥0.

The augmented Lagrangian function becomes


(6) $$\begin{array}{l} \;L=∥\left(A-UV^{T}\right){∥}_{F}^{2}+\frac{\lambda_{1}}{2}\left(∥U{∥}_{F}^{2}+∥V{∥}_{F}^{2}\right)\\ \;+\frac{\lambda_{2}}{2}\left(∥S_{d}-UP^{T}{∥}_{F}^{2}+∥S_{v}-VQ^{T}{∥}_{F}^{2}\right)\\ \;+\frac{\lambda_{3}}{2}\left(∥P{∥}_{F}^{2}+∥Q{∥}_{F}^{2}\right)+Tr\left(W^{T}\left(U-X\right)\right)\\ \;+Tr\left(R^{T}\left(U-X\right)\right)+\frac{\rho }{2}\left(∥U-X{∥}_{F}^{2}+∥V-Y{∥}_{F}^{2}\right) \end{array}$$

where W and R are the Lagrange multiplier and ρ>0 is the penalty parameter. At the i-th iteration, it requires alternatively computing $U_{i+1},V_{i+1},P_{i+1},Q_{i+1},X_{i+1},Y_{i+1},A_{i+1}$.

#### Molecular docking method

Molecular docking method can be used to study the behavior of small molecules at the binding sites of target proteins. It has been widely used in drug design, since structures of more and more target proteins have been confirmed by experiments. AutoDock (Goodsell, 1996) is an open source molecular simulation software available to identify the conformation of a small molecule binding to a large molecule target. AutoDock has an affinity scoring function, which can sort candidate poses according to the sum of van der Waals and electrostatic energy. We used AutoDock to evaluate the molecular binding activity between predicted antiviral drugs and biomolecules.

#### Evaluation metrics

In this work, we evaluate the predictive performance of our method by 5-fold cross-validation. Popular evaluation metrics: AUC and AUPR are used to quantify the predictive performance of methods. Given a threshold of predictive scores, the candidate associations above this threshold are regarded as positives, and others are negatives. Then, true positive rate (TPR), false positive rate (FPR) and Precision can be calculated by,


(7) TPR=TP/(TP+FN)

(8) FPR=FP/(FP+TN)

(9) Precision=TP/(TP+FP)

where TP, FP, TN, and FN represent true positive, false positive, true negative, and false negative, respectively. TPR is also called as Recall, which measures the ratio of correctly predicted positive samples to all positive samples. Precision measures the ratio of correctly predicted positive samples to all predicted positive samples.

With the increases of the threshold, TPR/Recall, FPR, and Precision will vary. TPR and FPR can form a TPR- FPR curve, called as the receiver-operating characteristic (ROC) curve. The area under the ROC curve is generally denoted as AUC. Precision and Recall (equivalent to TPR) can form a Precision–Recall (PR) curve. The area under the PR curve is generally denoted as AUPR. AUC and AUPR are scalar with the evaluation criterion: the larger AUC/AUPR is, the better the predictive performance is. AUC and AUPR can evaluate the overall performance of prediction algorithms.

## Results

### Parameter setting

In VDA-GKSBMF algorithm, there are tunable parameters $\gamma^{\prime },\omega ,\lambda_{1},\lambda_{2}\;\mathrm{and}\;\lambda_{3}\text{.}$ In order to prevent multi-parameter overfitting, we set $\lambda_{1},\lambda_{2}$ and $\lambda_{3}$ to the same value and remove two parameters. Because they are used to punish the related terms of U and V, P and Q in model (3) and model (4). VDA-GKSBMF has three parameters ($\gamma^{\prime },\omega ,\lambda_{1}$) needed to be determined. We first set $\gamma^{\prime }$ to 0.5, and then $\omega ,\lambda_{1}$ are set in range of {0, 0.1, 0.2,…, 1}, {0.001, 0.01, 0.1, 1} by using the fivefold cross-validation on the training dataset. Table 2 displays the top 3 AUCS values as a function of $\gamma^{\prime },\omega ,\lambda_{1},\lambda_{2}\;\mathrm{and}\;\lambda_{3}$ in five datasets.

**Table 2:** The top three AUCs using different $\gamma^{\prime },\omega ,\lambda_{1},\lambda_{2},\mathrm{and}\;\lambda_{3}$ values in 5-fold cross-validation.

Dataset	$\gamma^{\prime }$	ω	$\lambda_{1}$	$\lambda_{2}$	$\lambda_{3}$	AUC
Dataset1	0.5	0.3	1	1	1	**0.8851**
	0.5	0.4	1	1	1	0.8825
	0.5	0.5	1	1	1	0.8663
Dataset2	0.5	0.1	0.1	0.1	0.1	**0.8594**
	0.5	0.2	0.1	0.1	0.1	0.8590
	0.5	0.3	0.1	0.1	0.1	0.8583
Dataset3	0.5	0.4	1	1	1	**0.8807**
	0.5	0.3	1	1	1	0.8793
	0.5	0.2	1	1	1	0.8756
Dataset4	0.5	0.2	0.1	0.1	0.1	**0.8824**
	0.5	0.3	0.1	0.1	0.1	0.8809
	0.5	0.4	0.1	0.1	0.1	0.8766
Dataset5	0.5	0.4	1	1	1	**0.8804**
	0.5	0.3	1	1	1	0.8789
	0.5	0.5	1	1	1	0.8787

Bold represented the best AUC values of different parameters in the same datasets.

### Comparison with other methods

**Figure 2:**
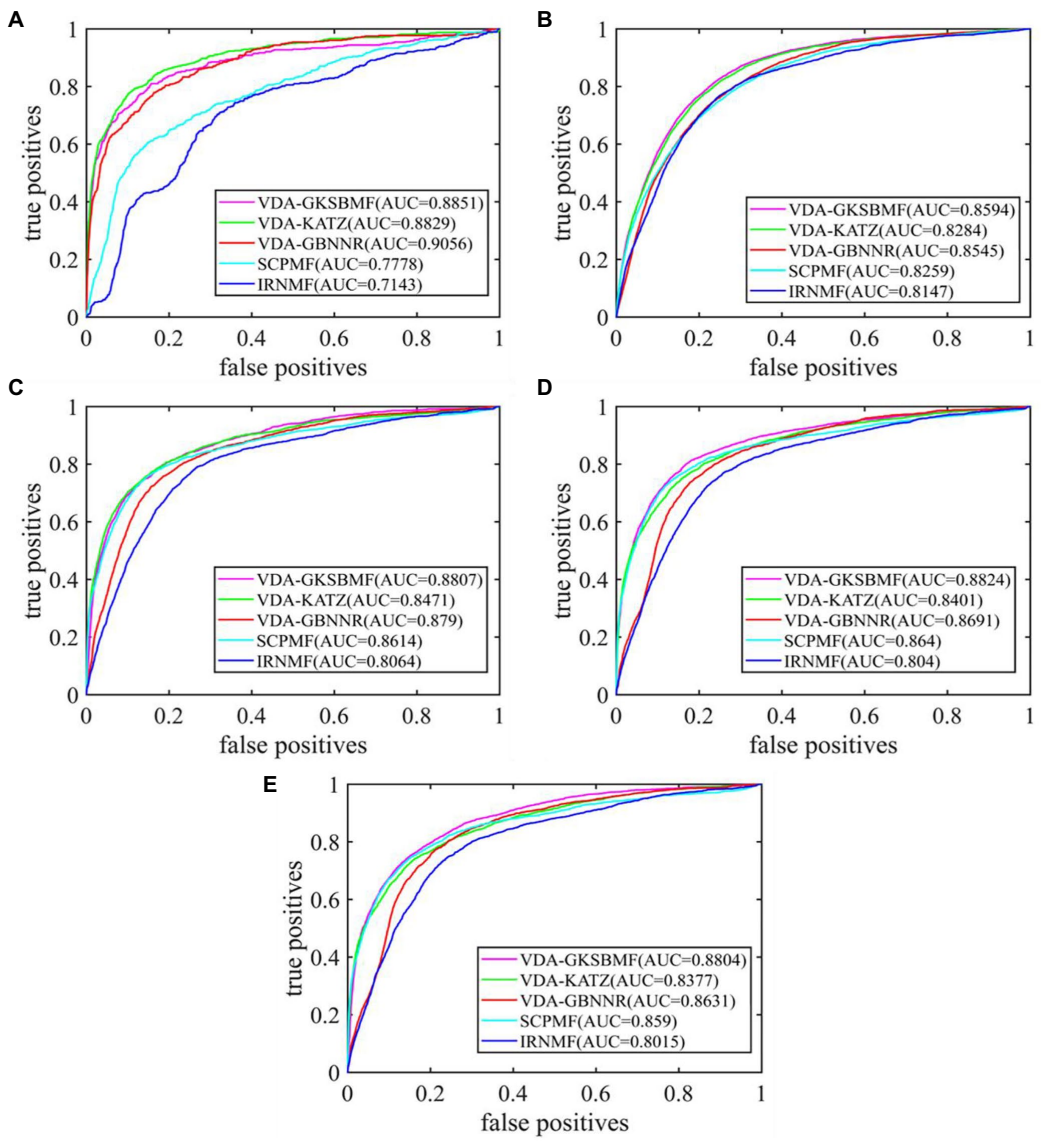
The performance of all methods in predicting virus–drug associations on five datasets: **(A)** Dataset1, **(B)** Dataset2, **(C)** Dataset3, **(D)** Dataset4, and **(E)** Dataset5.

By 5-fold cross-validation experiment, we evaluate the performance of VDA-GKSBMF. We plot its ROC curve in Figure 2, and we find that it has a high AUC value in five datasets.

Further, we compare the VDA-GKSBMF method with other methods for drug repositioning: VDA-KATZ (Yang et al., 2019), IRNMF (Tang et al., 2020), VDA-GBNNR (Wang et al., 2021), and SCPMF (Meng et al., 2021). VDA-KATZ (Yang et al., 2019) used a KATZ algorithm to infer drug-virus association. The Indicator Regularized non-negative Matrix Factorization (IRNMF) method (Tang et al., 2020) introduced the indicator matrix and Karush-Kuhn-Tucker condition into the non-negative matrix factorization algorithm. VDA-GBNNR based on kernel similarity to predict anti-SARS-COV-2 drug. SCPMF used similarity constrained probabilistic matrix to infer drug-virus association. The experiment was carried out 50 times, with average performance as the final result. Table 3 shows sensitivities, specificities, accuracies, and AUCs of the five models on the five datasets. From Table 3, VDA-GBNNR obtains the best performance for other methods in dataset 1. However, VDA-GKSBMF achieves the best sensitivity, accuracy, specificity, and AUC on dataset 2, dataset 3, dataset 4, and dataset 5. Figure 2 displays the results of the methods in five datasets. The results show that the VDA-GKSBMF method outperforms the baseline methods in terms of the ROC curves and the corresponding AUC values, meaning that it can better discover antiviral drugs.

**Table 3:** Performance indicators for different models.

Datasets	Methods	Accuracy	Sensitivity	Specificity	AUC
Dataset1	VDA-GKSBMF	0.5172	0.8757	0.5091	0.8851
	VDA-GBNNR	**0.5181**	**0.8957**	**0.5095**	**0.9056**
	VDA-KATZ	0.5171	0.8735	0.5090	0.8829
	SCPMF	0.5126	0.7708	0.5067	0.7778
	IRNMF	0.5098	0.7088	0.5052	0.7142
Dataset2	VDA-GKSBMF	**0.5136**	**0.8515**	**0.5072**	**0.8594**
	VDA-GBNNR	0.5134	0.8466	0.5071	0.8544
	VDA-KATZ	0.5125	0.8211	0.5066	0.8284
	SCPMF	0.5124	0.8187	0.5065	0.8259
	IRNMF	0.5120	0.8077	0.5063	0.8146
Dataset3	VDA-GKSBMF	**0.5097**	**0.8748**	**0.5052**	**0.8807**
	VDA-GBNNR	0.5097	0.8731	0.5051	0.8790
	VDA-KATZ	0.5089	0.8416	0.5047	0.8471
	SCPMF	0.5093	0.8557	0.5049	0.8613
	IRNMF	0.5079	0.8015	0.5042	0.8063
Dataset4	VDA-GKSBMF	**0.5102**	**0.8763**	**0.5054**	**0.8824**
	VDA-GBNNR	0.5098	0.8631	0.5052	0.8691
	VDA-KATZ	0.5091	0.8345	0.5048	0.8400
	SCPMF	0.5097	0.8581	0.5051	0.8639
	IRNMF	0.5081	0.7990	0.5044	0.8040
Dataset5	VDA-GKSBMF	**0.5101**	**0.8743**	**0.5054**	**0.8804**
	VDA-GBNNR	0.5096	0.8572	0.5051	0.8630
	VDA-KATZ	0.5090	0.8322	0.5048	0.8376
	SCPMF	0.5095	0.8532	0.5051	0.8590
	IRNMF	0.5081	0.7966	0.5043	0.8015

Bold represented the best value of different methods under the same evaluation condition.

### Case study

After verifying the good performance of VDA-GKSBMF, to discover unknown antiviral drugs against SARS-CoV-2, we predict potential associations between SARS-CoV-2 and small molecule drugs based on known drug-virus association data, and we obtain the top-10 drugs with the highest score (see Table 4) in five datasets. Among the top-10 predicted drugs, there are 10 drugs that have been reported in the relevant literature, but the small molecule drugs were never confirmed to be anti-SARS-CoV-2 antiviral drugs. Ribavirin, Remdesivir, Oseltamivir, and Zidovudine were existed in at least four datasets.

**Table 4:** The predicted top-10 antiviral drugs against SARS-CoV-2 in five datasets.

Dataset1-drug	Dataset2-drug	Dataset3-drug	Dataset4-drug	Dataset5-drug
**Remdesivir**	Favipiravir	**Ribavirin**	Nitazoxanide	**Ribavirin**
**Oseltamivir**	**Remdesivir**	Nitazoxanide	**Ribavirin**	Chloroquine
Zanamivir	Cidofovir	Chloroquine	**Oseltamivir**	**Zidovudine**
**ribavirin**	**ribavirin**	Camostat	Camostat	Camostat
Laninamivir	Mycophenolic acid	Umifenovir	**Zidovudine**	Umifenovir
Peramivir	Navitoclax	**Remdesivir**	Favipiravir	Favipiravir
Presatovir	Itraconazole	**Zidovudine**	Hexachlorophene	Rifamycin
**zidovudine**	BCX4430 (Galidesivir)	Berberine	**Remdesivir**	**Oseltamivir**
Mycophenolic acid	Pleconaril	Amantadine	Sirolimus	Berberine
Mizoribine	Cyclosporine	**Oseltamivir**	Suramin	Niclosamide

Bold indicated that the drug existed in at least four datasets.

Ribavirin is a road-spectrum antiviral drug that can inhibit the replication of respiratory syncytial virus (van Laarhoven and Marchiori, 2013). It can prevent respiratory syncytial virus infection in lung transplant recipients, and has been used to treat SARS-CoV and MERS-CoV. Similar to SARS-CoV and MERS-CoV, SARS-CoV-2 are a respiratory syndrome beta coronavirus that may cause severe respiratory diseases, and a few studies have reported that ribavirin may take an inhibitory effect on SARS-CoV-2 (Peng et al., 2020).

Remdesivir is a nucleoside analog with antiviral activity. Remdesivir has broad-spectrum activities against RNA viruses, such as SARS and MERS, and has been studied in a clinical trial for Ebola.

Oseltamivir is an antiviral neuraminidase inhibitor (Oseltamivir, n.d.) and has been used to prevent the infection of influenza A virus (for example, A-H1N1; Meijer et al., 2009, A-H5N1; De Jong et al., 2005, and influenza B virus). Oseltamivir can prevent the germination, replication, and infectivity of the virus in the host cell. More importantly, Oseltamivir combined with other drugs has been reported to inhibit the infection of SARS-CoV-2 (Huang et al., 2020).

### Molecular docking

To further study the effectiveness of predicted drugs against SARS-CoV-2, the top 10 predicted small molecules are molecularly docked with SARS-CoV-2 spike protein/ACE2. From the DrugBank database, the chemical structures of these small molecule drugs have been obtained. The structure of spinous process protein of SARS-CoV-2 is calculated based on the homology model of Zhang lab (Wang et al., 2020). We used AutoDock, a bioinformatics tool, to conduct molecular docking between the predicted antiviral drug and SARS-CoV-2 spike protein/ACE2. The search algorithm scans the entire protein in AutoDock by genetic algorithm and grid box.

We calculate the predicted molecular binding energies of ribavirin, remdesivir, oseltamivir, and zidovudine small molecules with the spinous process protein and ACE2 of SARS-CoV-2 in Table 5. The results show that the binding activities of ribavirin with these two proteins are −5.29 and −6.39 kcal/mol, followed by remdesivir with −5.22 and −7.4 kcal/mol, and oseltamivir with −4.04 and − 4.73 kcal/mol. More importantly, ribavirin and remdesivir have been used to treat SARS, and their sequence homology with SARS-CoV-2 is about 79%.

**Table 5:** The molecular binding energies between the predicted 4 antiviral drugs and two target proteins at least four datasets.

Drugs	Binding energies of target proteins	
	Spike protein	ACE2
Ribavirin	−5.29	−6.39
Remdesivir	−5.22	−7.40
Oseltamivir	−4.04	−4.73
Zidovudine	−6.54	−7.93

Zidovudine has molecular binding energies of −6.54 and − 7.93 kcal/mol. Zidovudine is the drug which is an effective HIV replication inhibitor, which can improve immune function and partially reverse the neurological dysfunction caused by HIV. zidovudine, as an HIV nucleoside/nucleotide analogues reverse transcriptase inhibitor, has the potential to be a clue for SARS-COV-2 treatment.

**Figure 3:**
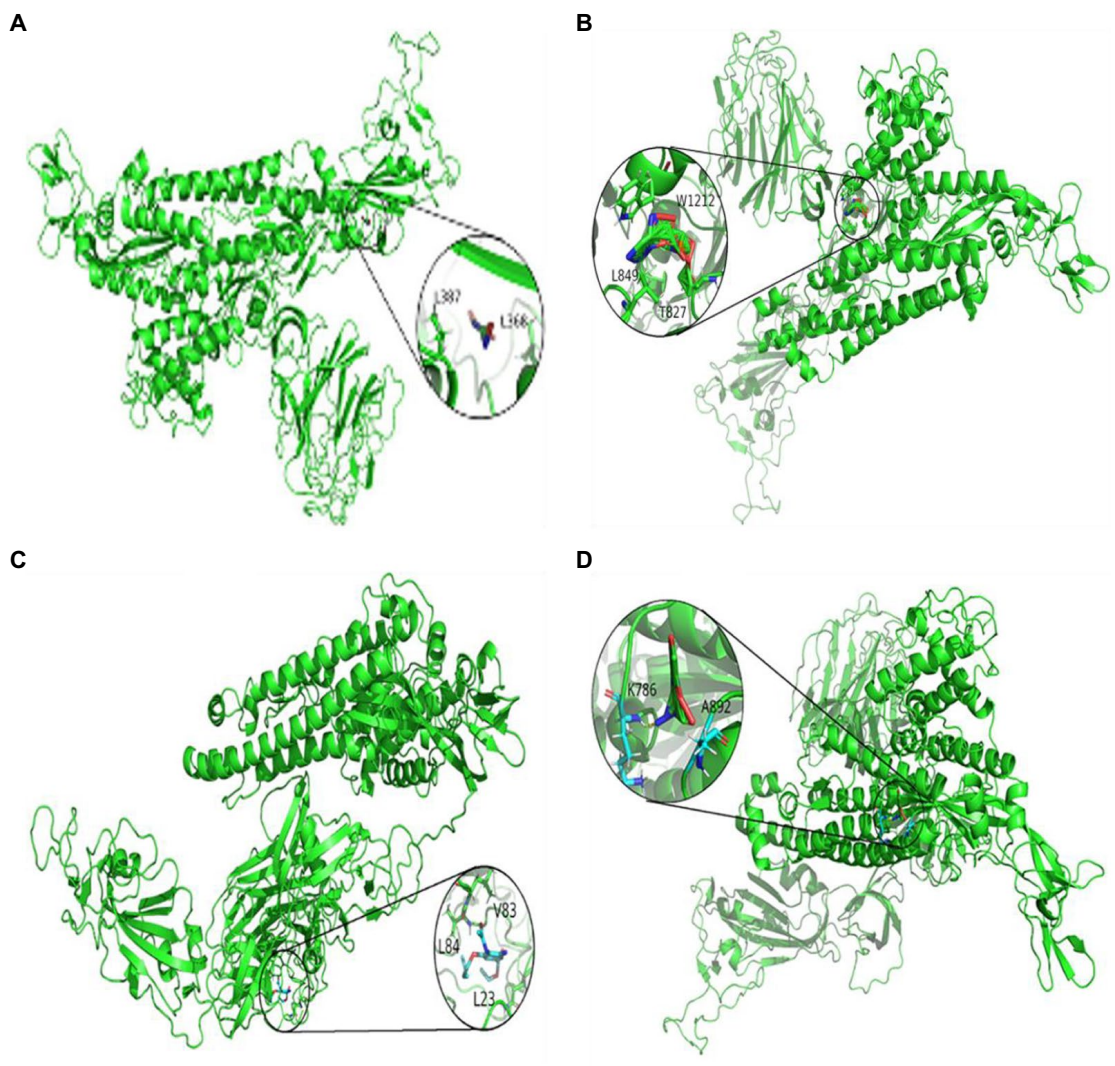
Molecular docking between the spike protein and four drugs: **(A)** ribavirin, **(B)** remdesivir, **(C)** oseltamivir, and **(D)** zidovudine.

**Figure 4:**
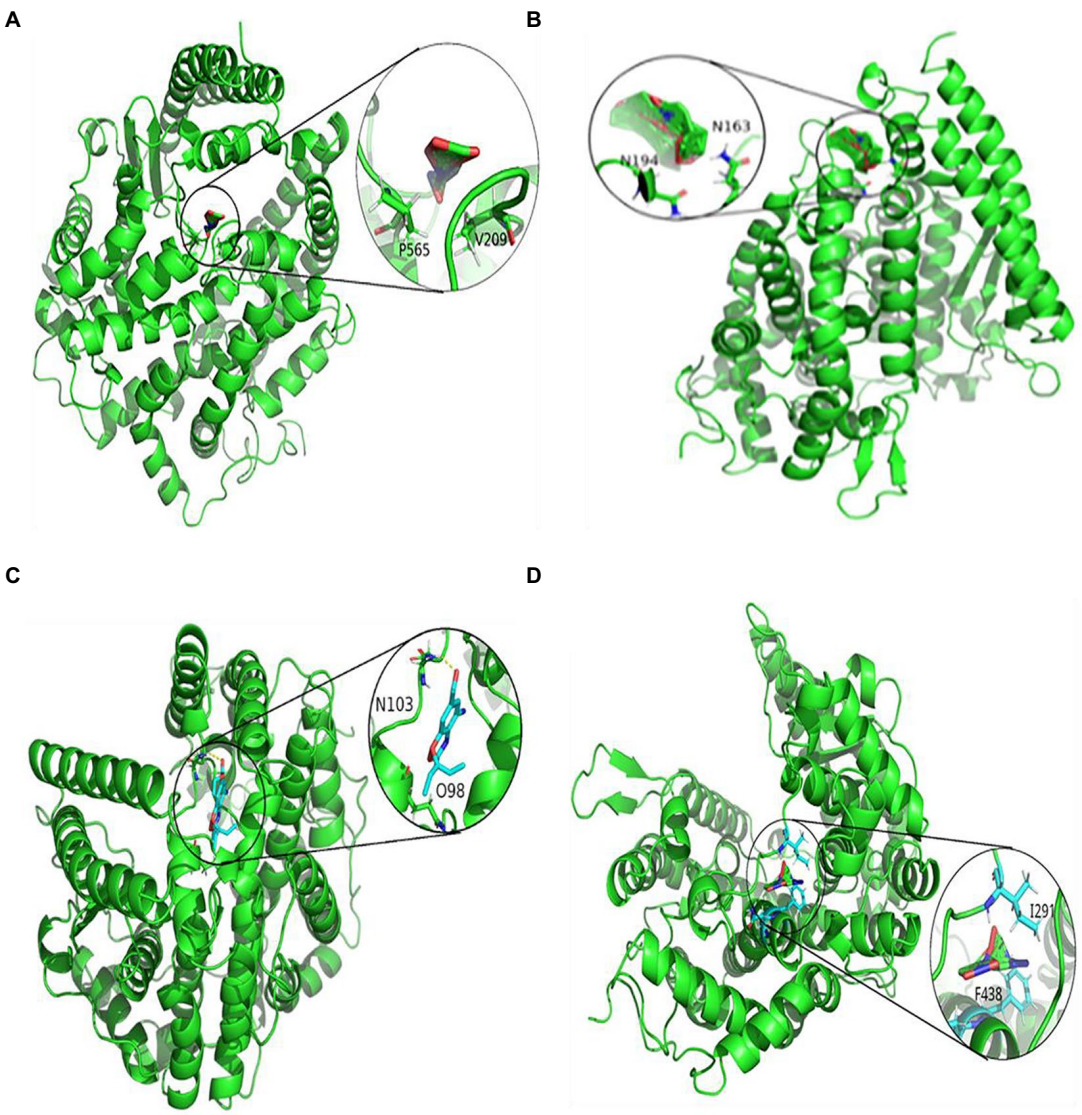
Molecular docking between ACE2 and four drugs: **(A)** ribavirin, **(B)** remdesivir, **(C)** oseltamivir, and **(D)** zidovudine.

Figures 3, 4 represent the docking results of four small molecules including ribavirin, remdesivir, oseltamivir, and zidovudine with two target proteins. The circles in each subgraph indicate the binding sites of the drug to the target protein. For example, the amino acids L387, L368, P565, and V209 are inferred to be the key residues for ribavirin binding to the SARS-CoV-2 spike protein/ACE2, while L849, T827, W1212, L144, and P504 are predicted as the key residues for remdesivir binding to these two target proteins.

## Discussion

Severe acute respiratory syndrome coronavirus 2 is quickly diffusing throughout the world, and it is urgent to find effective treatments against this virus. Drug repositioning, seeking to find new uses, offers a new strategy for the treatment of SARS-COV-2. However, to date, only a few databases have collated relevant drugs that may be used to treat SARS-COV-2. Thus, we developed a drug-virus as well as a method VDA-GKSBMF to prioritize drugs against SARS-COV-2.

Specifically, VDA-GKSBMF has a high AUC in cross-validation, which is better than other state-of-art methods in four datasets. We measured the molecular binding activity between predicted antiviral drugs and SARS-CoV-2 spike protein/human ACE2 (Zhao et al., 2020). Among them, the molecular binding energies between ACE2 and the four drugs were: Ribavirin (−6.39 kcal/mol), Remdesivir (−7.4 kcal/mol), Oseltamivir (−4.73 kcal/mol), zidovudine (−7.93 kcal/mol), and the four drugs have been in clinical trials or supported in recent publications. The results suggest that the VDA-GKSBMF algorithm can effectively infer unknown drugs of SARS-COV-2.

However, there a few limitations of this study. First, due to the limited size of the current virus-drug dataset and the complexity of intrinsic relationship in biomedical data, VDA-GKSBMF still has room for further improvement. On the one hand, we would like to expand the virus-drug dataset by including more virus-related and drug-related information, so as to further improve the predictive power of mining hidden virus-drug associations. On the other hand, it is also possible to enhance the ability of discovering potential drugs against SARS-COV-2 by more advanced and methods in related fields (Xu et al., 2020b; Xiang et al., 2021b, 2022a; Meng et al., 2022). Second, though we performed literature mining and molecular docking to validate our results, they are all in-silico methods. The prioritized drugs should be validated using wet-lab experiments. However, it is out of the scope of this study.

## Conclusion

In this study, we collected five virus-drug datasets including VDAs matrix, virus genomic sequence similarity matrix, and drug chemical structure similarity matrix and explored drug repositioning of SARS-COV-2 by a novel method called VDA-GKSBMF.VDA-GKSBMF combined Gaussian similarity and extracted useful features to deduce potential virus-drug associations. It combined Gaussian similarity and virus-drug association into the target function. The non-negative constraint was used in VDA-GKSBMF, ensuring that the predicted scores of association matrix were non-negative for the biological interpretability. Our results showed that VDA-GKSBMF is an effective approach for discovering new drugs of SARS-COV-2. In the future, we will combine different data resources to create larger dataset and design integrated algorithm, integrating multiple heterogeneous network and multiple similarities for predicting potential virus-drug associations.

## Data availability statement

Publicly available datasets were analyzed in this study. This data can be found at: https://github.com/xiangju0208/VDA_GMSBMF.

## Author contributions

BH and JH contributed to conception and design of the study. YW and JX organized the data and the prediction model. MT, RH, CL, and GT performed the statistical analysis. YW, JX, MB, JH, and BH wrote the manuscript. All authors contributed to the article and approved the submitted version.

## Funding

This study was supported by the Training Program for Excellent Young Innovators of Changsha (Grant Nos. kq1802024, kq1905045, kq2009093, and kq2106075), Hunan key laboratory cultivation base of the research and development of novel pharmaceutical preparations (No. 2016TP1029), Hunan Provincial Innovation Platform and Talents Program (No. 2018RS3105), the Foundation of Hunan Educational Committee (Grant No. 19A060), and the Provincial key R & D projects of Hunan Provincial Science and Technology Department (No. 2022SK2074). This research was funded by the Natural Science Foundation of Hunan province (No. 2018JJ2461), the Project to Introduce Intelligence from Oversea Experts to Changsha City (Grant No. 2089901), and General project of Education Department of Hunan Province (Grant No. 19C0190), and supported by the special fund of “Young and Middle-aged Key Teachers Training Program” of Changsha Medical College, the National Natural Science Foundation of China (32002235).

## Conflict of interest

RH and GT are employed by Genesis (Beijing) Co. Ltd.

The remaining authors declare that the research was conducted in the absence of any commercial or financial relationships that could be construed as a potential conflict of interest.

## Publisher’s note

All claims expressed in this article are solely those of the authors and do not necessarily represent those of their affiliated organizations, or those of the publisher, the editors and the reviewers. Any product that may be evaluated in this article, or claim that may be made by its manufacturer, is not guaranteed or endorsed by the publisher.
